# Self-Calibration Technique with Lightweight Algorithm for Thermal Drift Compensation in MEMS Accelerometers

**DOI:** 10.3390/mi13040584

**Published:** 2022-04-08

**Authors:** Javier Martínez, David Asiain, José Ramón Beltrán

**Affiliations:** 1 Department of Electronic Engineering, Escuela Universitaria Politécnica de la Almunia, C/Mayor 5, La Almunia de Doña Godina, 50100 Zaragoza, Spain; dasiain@unizar.es; 2 Department of Electronic Engineering and Communications, Universidad de Zaragoza, C/María de Luna 1, 50018 Zaragoza, Spain; jrbelbla@unizar.es

**Keywords:** MEMS, accelerometer, thermal drift, thermal compensation, calibration technique

## Abstract

Capacitive MEMS accelerometers have a high thermal sensitivity that drifts the output when subjected to changes in temperature. To improve their performance in applications with thermal variations, it is necessary to compensate for these effects. These drifts can be compensated using a lightweight algorithm by knowing the characteristic thermal parameters of the accelerometer (Temperature Drift of Bias and Temperature Drift of Scale Factor). These parameters vary in each accelerometer and axis, making an individual calibration necessary. In this work, a simple and fast calibration method that allows the characteristic parameters of the three axes to be obtained simultaneously through a single test is proposed. This method is based on the study of two specific orientations, each at two temperatures. By means of the suitable selection of the orientations and the temperature points, the data obtained can be extrapolated to the entire working range of the accelerometer. Only a mechanical anchor and a heat source are required to perform the calibration. This technique can be scaled to calibrate multiple accelerometers simultaneously. A lightweight algorithm is used to analyze the test data and obtain the compensation parameters. This algorithm stores only the most relevant data, reducing memory and computing power requirements. This allows it to be run in real time on a low-cost microcontroller during testing to obtain compensation parameters immediately. This method is aimed at mass factory calibration, where individual calibration with traditional methods may not be an adequate option. The proposed method has been compared with a traditional calibration using a six tests in orthogonal directions and a thermal chamber with a relative error difference of 0.3%.

## 1. Introduction

Microelectromechanical (MEMS) inertial sensors are increasingly being implemented in consumer electronics as the preferred movement detection technology. Their advantages over traditional technologies include lower cost, smaller size, and lower energy consumption, making them the ideal solution for many electronic products.

The massive implementation of inertial systems in smartphones, among other electronic products, has allowed multiple studies to be carried out, such as user transportation means detection [1], pedestrian recognition [2], or structural integrity monitoring [3].

Their use in measurement and research applications is not limited to the implementation in consumer electronics, and they are currently being used in multiple fields such as railway infrastructure [4], geotechnical monitoring [5], geostructural safety [6], surface failure of slopes [7], bridge structural monitoring [8], and even tree property measurements [9].

However, MEMS inertial sensors have some disadvantages compared to traditional technologies. One major drawback, which can become significant in some cases, is the thermal dependence these sensors show. This dependence reduces its suitability for outdoor or thermally variable applications. The drift in the output value caused by this thermal sensitivity is not deterministic, since each unit and axis exhibits this behavior to varying degrees [10,11]. Therefore, the thermal behavior of each unit has to be studied and compensated individually.

There are two main approaches to reduce the thermal drifts in MEMS sensors: hardware solutions and software solutions. Hardware solutions aim to actively reduce the effects of the thermal drift. This can be achieved with alternative designs of the sensing structure [12,13,14,15,16], including additional electronics [17,18,19,20], or isolating the device from external temperatures [21]. However, these techniques usually increase the cost or complexity of the system, and since they are built-in features, they have to be planned in advance. These techniques can enhance the capabilities of the technology, but in order to reduce the thermal drift of an existing accelerometer in application, other methods are required.

The main approach to compensate for the thermal drift in existing sensors and systems relies on modeling the thermal behavior and compensating its effects after the data have been captured. Second-order surfaces [22], third-order curves [23], and neural networks [24] have been used to compensate for thermal drifts in previous works. However, to properly fit the polynomial parameters, or the neural weights, the system’s behavior is usually studied in its full working ranges for temperature and acceleration. With these techniques, the time and cost required to calibrate each sensor increases with each additional degree of the polynomial model or with each neuron added. Six or more tests are usually required for the calibration, each one taking multiple hours to complete. Generally, external hardware and software is also needed to compute the calibration parameters. The requirement of individual calibration can increase significantly the cost in industrial or mass production environments when using these techniques.

The objective of this work is to propose a fast and low-cost thermal calibration technique for MEMS inertial sensors. This calibration technique is able to obtain the compensation parameters without complex laboratory equipment or software, similar to in-field calibration techniques for bias and sensitivity [25]. A lightweight algorithm is developed to store only the most relevant data, reducing the memory requirements, and then compute the calibration parameters. This can be especially useful in industrial environments with mass productions, where individual calibration is not viable. The thermal compensation technique used is based on the model with the TDB (Thermal Drift of Bias) and TDSF (Thermal Drift of Scale Factor) parameters [11] due to its reduced amount of calibration parameters.

This paper is organized as follows. Initially, Section 2 presents an overview of the MEMS accelerometer, describing its working principle and the thermal behavior. Section 3 presents the proposed calibration technique, including the self-calibration algorithm. Section 4 presents the proposed methodology and testing conditions. Section 5 shows the tests results, the algorithm analysis, and the compensation performance. Finally, Section 6 highlights the conclusions obtained from this work and the potential use of the proposed technique.

## 2. The MEMS Accelerometer

**Figure 1:**
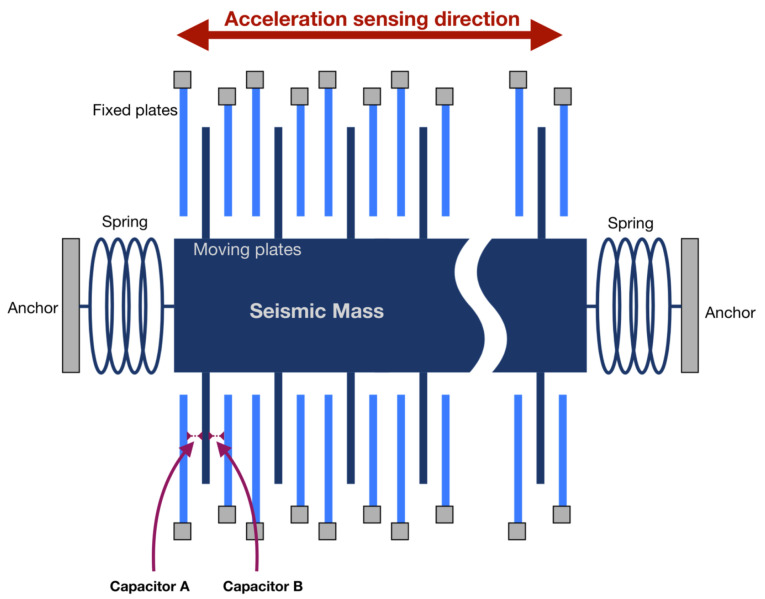
Working principle of a capacitive accelerometer.

MEMS capacitive accelerometers are based on a spring-mass system. Any external acceleration produces a displacement in the mass. Using opposed plated capacitors with one plate in the mass and the other fixed, this displacement is related to a capacitance variation [26]. The difference in capacitance between pairs of capacitors can be used to determine the external acceleration. A diagram of this structure is shown in Figure 1.

Two identical structures can be manufactured perpendicularly to obtain a biaxial accelerometer; the third axis, usually Z, requires an alternative sensing technique [26]. This results in different characteristics for this third axis, which generally suffers from lower sensitivity and worse performance.

The thermal drift has been linked in some studies to manufacturing imperfections in the sensing structures [27], all of which are made of silicon. Silicon is a temperature-sensitive material; therefore, temperature affects the internal structures in multiple ways: Young modulus variation, deformations, and stresses [28]. Temperature variations, such as those typical of consumer, industrial, or outdoor applications, can induce temporal or permanent stresses in the micromechanical structures. These can affect both the zero level and the sensitivity of the sensor, resulting in a significant drift of the output value. Thermal variations can be produced by both external and internal phenomena, such as ohmic losses.

This thermal behavior can be modeled using two parameters: the Thermal Drift of Bias and the Thermal Drift of Scale Factor [16,29,30]. The TDB, expressed in mg/°C, has the largest impact, and it is random in both value and sign. The TDSF, expressed in ppm/°C, has a lower impact on the thermal drift and is always negative. Manufacturers of MEMS inertial systems usually provide data about the typical values of these parameters. They often consider 25 °C as a reference temperature for all the measurements, as that is the temperature where the thermal drift is supposed to be zero. Previous studies suggest that this thermal drift can reach up to ±1.5 mg°C, making it necessary to compensate for its effects in any medium to high-precision application.

According to the TDB and TDSF model, as shown in Equation (1), the output value of the accelerometers, Acc, depends on the real acceleration, $Acc_{0}$; the internal temperature, *T*; the reference temperature, $T_{R}$; and both thermal drift parameters, TDB and TDSF.

(1) $$Acc=Acc_{0}+\left(T-T_{R}\right)\left(TDB+TDSF\cdot Acc_{0}\right)$$

## 3. Proposed Technique

According to that thermal model, the thermal drift for a static orientation has to be proportional to the thermal variation. Furthermore, this thermal drift for a specific orientation (*i*), which will be called $TD_{i}$ from now on, should be equal to the TDB plus the TDSF times the real acceleration in that orientation ($Acc_{0i}$), as shown in Equation (2). Since the $TD_{i}$ does not depend on the temperature value, it should remain constant for any given temperature, and therefore, it can be computed as the ratio between any temperature variation and its related acceleration drift, as shown in Equation (3). Knowing multiple $TD_{i}$ and their respective accelerations, it is possible to obtain the TDB and TDSF values. The real acceleration can be computed, using the $TD_{i}$, as the acceleration at the reference temperature, as shown in Equation (4). This reference temperature is set to 25 °C, according to manufacturers and previous studies. Since two parameters have to be obtained (TDB and TDSF), two $TD_{i}$ with their respective $Acc_{0}$ points are required. However, each pair of $TD_{i}$ and $Acc_{0i}$ requires at least two temperatures to be computed. The further apart these temperatures are, the greater the signal-to-noise ratio of the system becomes, increasing the accuracy of the compensation technique.

(2) $$TD_{i}=TDB+TDSF\cdot Acc_{0i}$$

(3) $$TD_{i}=\frac{\Delta Acc_{i}}{\Delta T_{i}}$$

(4) $$Acc_{0i}=Acc_{i}-TD_{i}\cdot \left(T_{i}-25\right)$$

To carry out the calibration tests, one simple option is to align the gravity vector to each axis, having two separate tests for each axis. One faster alternative, which will be used in this work, consists of using just two orientations to calibrate all three axes simultaneously. This solution reduces the amount of required calibration tests for a triaxial accelerometer from six to two.

Therefore, the technique will consist of two mechanical orientations, two temperatures, and one algorithm for self-calibration.

### 3.1. Mechanical Orientations

Since the calibration will be performed using only two orientations for all three axes, they should ensure the maximum working range for all three axes simultaneously. Gravity (*g*) will be used as the only external force acting on the system; therefore, the maximum working range is achieved when the acceleration magnitude in all axes is equal. The acceleration in each individual axis can be computed using Equation (5). Afterwards, the orientation of the sensor can be described by computing the Euler angles using Equations (6) and (7).

(5) $$Acc_{X}=Acc_{Y}=Acc_{Z}=Acc_{0}\to g^{2}=3\cdot Acc_{0}^{2}\to Acc_{0}=\pm 0.577g$$

(6) $$\alpha =\arccos\left(\frac{Acc_{Z}}{g}\right)=55^{\circ }$$

(7) $$\beta =\arcsin\left(\frac{Acc_{Y}}{g\cdot \sin(\alpha )}\right)=45^{\circ }$$

**Figure 2:**
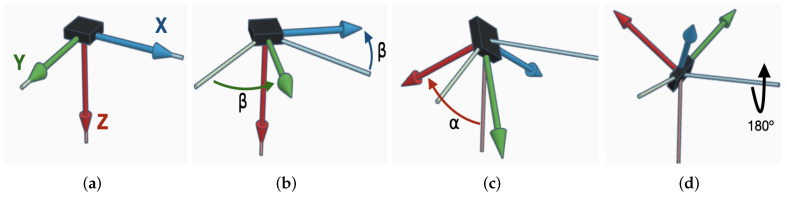
Rotation sequence used during the calibration tests. (**a**) Reference Orientation. (**b**) First Rotation. (**c**) Positive Forces Orientation. (**d**) Negative Forces Orientation.

These Euler angles computed correspond to the orientation where every axis reads a positive acceleration of 0.577 *g*. The sequence of orientations required for the test is shown in Figure 2. Starting from the reference axes (Figure 2a), a 45° rotation along the Z axis in red and 55° rotation along the Y axis in green (Figure 2b) result in first test orientation (Figure 2c). To obtain negative forces in all axes, corresponding to the second orientation (Figure 2d), a 180° rotation along the X axis, in blue, is performed.

No external references, such as other accelerometers or tilt measurements, are used during the calibrations, only the data obtained from the Device Under Test (DUT). The thermal calibration should be performed prior to the mechanical calibration, since the thermal calibration only affects each sensing axis individually. In contrast, after the mechanical calibration, the output values represent the acceleration along the reference axes and are affected by the three sensing axes simultaneously. Not being mechanically calibrated means that the external references would not match the actual data due to misalignments, bias deviations, or sensitivity deviations. However, any imperfection in the calibration angles should not affect the calibration in a significant way, since the data used for calibration are exclusively provided by the DUT. At the same time, large deviations in these orientations could still affect the calibration performance in case of a drastic reduction of the study range.

### 3.2. Thermal Requirements

To ensure an adequate analysis of the thermal drifts, it has to be distinguishable from noise and other effects; the larger the thermal variations, the easier it will become. Two different temperatures are needed; the ambient temperature can be used as the first temperature, thus, only one extra temperature is required. This second temperature is decided to be hotter than the ambient temperature, since a heat source is easier to generate than a cold source.

Thermal variations are usually slow and largely responsible for the duration of calibration tests. The proposed technique does not take into account the system’s behavior during the thermal transition. Therefore, this transition can be shortened by increasing the thermal gradient, thus significantly reducing the duration of the tests.

In our case, it was decided that the heat source has to increase the temperature by at least 20 °C. To generate these thermal variations on the DUT, a contactless method is preferred, since this helps to avoid mechanical stresses by contact. This should leave the thermal drift as the only effect that affects the output value of the accelerometer.

### 3.3. Self-Calibration Algorithm

**Figure 3:**
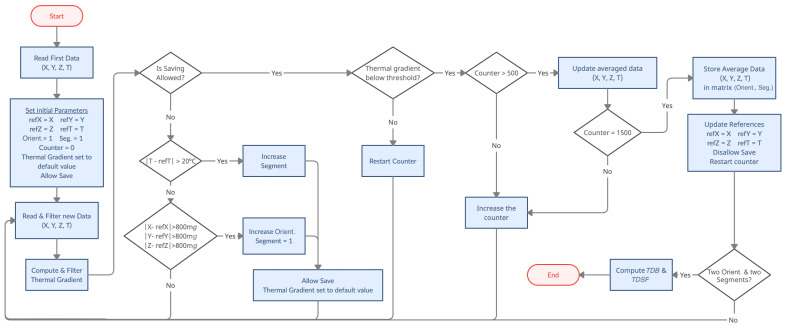
Working flow of the proposed algorithm.

The algorithm used to obtain the thermal characteristic parameters has to be able to be executed in real time by a wide range of microcontrollers, including those with limited resources. Therefore, two main aspects need to be taken into account: the computing time and the memory requirements. In this regard, the proposed algorithm analyzes the data sequentially, minimizing the number of operations per cycle, and stores just a few representative values instead of the full tests. The computation of the calibration parameters is performed when the test ends using only the averaged data and does not require complex calculations. The working flow of the data acquisition part of the algorithm is shown in Figure 3.

Initially, all measurements are filtered using an exponential filter with α=0.05. Then, the thermal gradient is computed and filtered again, this time with α=0.01. This increases the response time of the system and ensures the thermal stability in the segments that are logged. When the thermal gradient is below a certain threshold, 1 °C per minute, the software is allowed to store the data. However, the first 500 data are discarded to further ensure data stability. After this segment, which is restarted if the gradient threshold is exceeded, 1000 measures are captured and averaged for all the axes and temperature. At this point, the algorithm stops logging and waits for a change in temperature or orientation. Any thermal variation greater than 20 °C indicates a segment change. The orientation change is registered when all axes detect a deviation larger than 0.8 *g* from the last saved data. Both the segment and the orientation changes reset the gradient value to a high value, avoiding any false logging due to slow response time. The algorithm requires at least two logged segments in both orientations to proceed with the TDB and TDSF computation.

When the fast calibration test ends, the algorithm will have generated four matrices containing all the relevant information, one for each axis and one for the temperature. Each cell in each matrix represents the average value in a specific orientation and temperature segment, as shown in Table 1. The algorithm only requires two temperature steps in each orientation, although more can improve the performance.

One thermal drift coefficient is obtained for every two temperatures in the same orientation using Equation (3). The theoretical acceleration is also computed for each pair of steps using Equation (4). When multiple $TD_{i}$s and $Acc_{0i}$s are obtained for the same test orientation, the averages are computed. This happens when one orientation is analyzed with more than two temperatures. After this, the algorithm stores three matrices, one per axis, with four values each, two theoretical accelerations, and their corresponding thermal drifts (see Table 2).

Finally, the characteristic parameters TDSF and TDB are computed for each axis using Equations (8) and (9), respectively.

(8) $$TDSF=\frac{TD_{1}-TD_{2}}{Acc_{01}-Acc_{02}}$$

(9) $$TDB=TD_{1}-TDSF\cdot Acc_{01}$$

The computed values can suffer some deviation from the real values due to the system’s noise. This can affect the TDSF more easily, since its value is much lower and, therefore, more sensitive to noise. However, since this value should always be negative, any positive value for the TDSF is converted to zero at the end of the algorithm.

## 4. Methodology

### 4.1. Test Equipment

To achieve the two test orientations, a simple mechanical structure is proposed. It ensures the correct angles in both orientations and allows for an easy turn from one to another. The proposed solution consists of a metal plate placed on two supports at different heights, generating the 55° pitch. This plate can be inverted to generate the second orientation without moving the supports. The 45° angle is achieved with a rotation between the metal plate and the DUTs. To minimize the propagation of mechanical stresses from the calibration structure to the printed circuit board, and therefore the DUT, only one anchor point is advised.

The heat source chosen for this work consists of an incandescent infrared light. This results in a directed heat source that generates no force on the sensor. The alternative could be using hot air, but this has the disadvantages of being slow or requiring a concentrated air flow, which could result in forces being transmitted to the accelerometer. The lamp is attached to the metal plate; this way, its relative position to the DUT remains constant during the tests.

**Figure 4:**
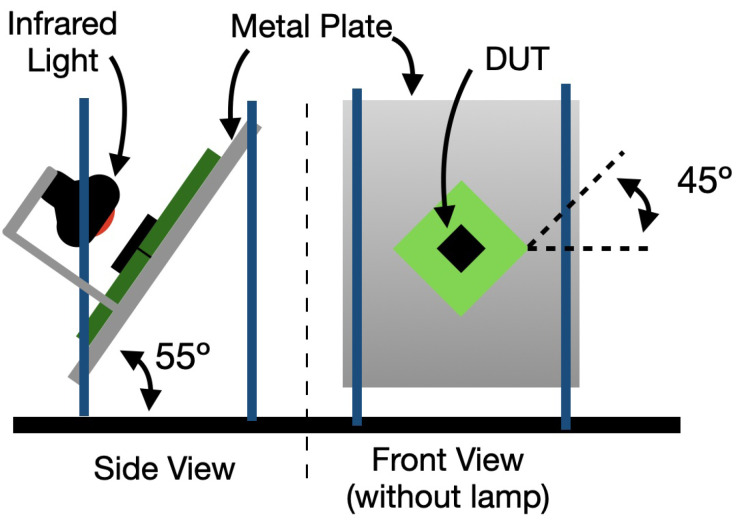
Proposed mechanical structure to perform the calibration tests.

One simplified diagram of the proposed structure is shown in Figure 4.

### 4.2. Device Under Test

To test this technique, the LIS3DSHTR MEMS accelerometer, manufactured by STMicroelectronics (Geneva, Switzerland), is used. It is a triaxial capacitive accelerometer with low cost, size, and energy consumption, which encourages its use in Internet of Things systems, portable devices, and mass productions. This device also includes an internal 8 bit temperature sensor, removing the need for an external temperature sensor, which would not accurately measure the internal temperature. The technical specifications and typical characteristics of this accelerometer are shown in Table 3. The configuration chosen during the tests is shown in Table 4.

**Figure 5:**
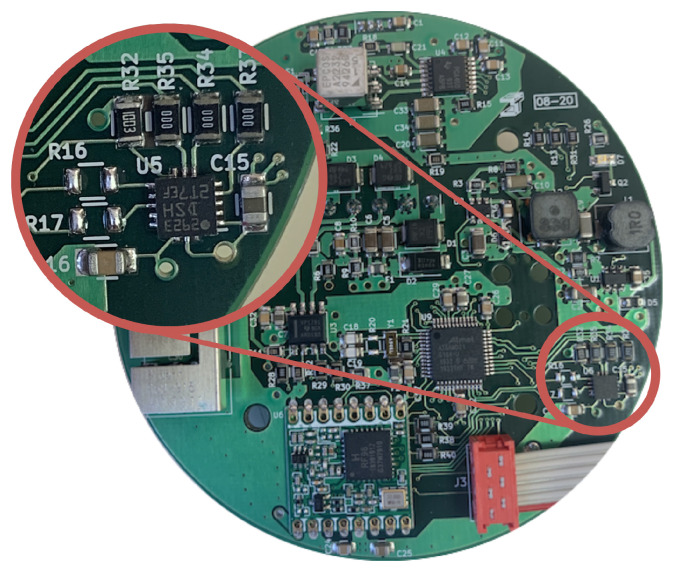
Board used during the calibration tests.

This sensor is integrated in a printed circuit board with the SAMD21G18A microcontroller (MCU), manufactured by Microchip Technology Inc. (Chandler, AZ, USA). This MCU runs the self-calibration algorithm and acts as the interface between the sensor and a computer logging the data. Power supply and communications electronics are also integrated in the board. This can increase the self-heating effect during the first minutes of the tests, similarly to how any application board would do. The test board, and a detail of the accelerometer, is shown in Figure 5.

It is also worth noting that due to the energetic consumption of the sensor, its internal temperature will rise slightly after power-up. This effect has to be taken into account to perform the thermal calibrations. The proposed algorithm avoids this first region, since it does not take into account any data while the thermal gradient exceeds a certain threshold.

### 4.3. Test Conditions

**Figure 6:**
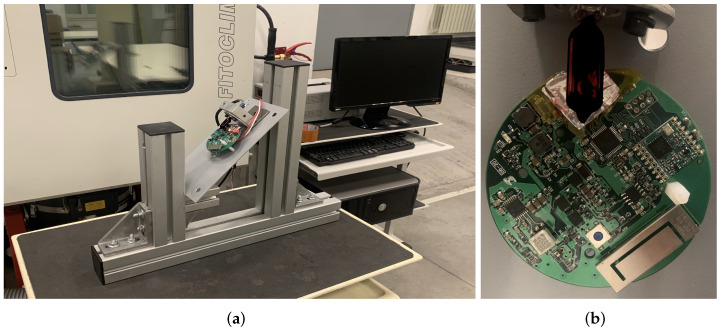
Hardware used during the tests. (**a**) Calibration structure (front), thermal chamber (left), and logging pc (right). (**b**) DUT with the cover ready to be tested.

Four identical units are tested to ensure the actual performance of this technique. All of them have been produced with the same industrial process. The accelerometer is covered with two layers of kapton and tinfoil tapes to ensure a proper thermal uniformity in the sensor and its pads. Unwanted behaviors have been noticed in the sensor if left uncovered; this is believed to be due to irregular heating in the area. The kapton and tinfoil cover stabilizes the thermal behavior of the accelerometer without increasing the thermal inertia of the system. Figure 6 shows the DUT ready to be tested in the calibration tooling.

**Figure 7:**
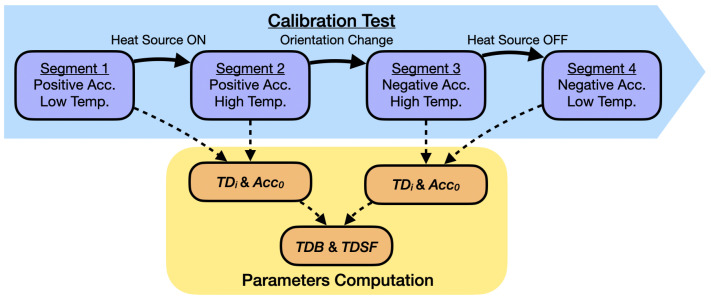
Working flow of the calibration technique.

The tests starts in the orientation with positive acceleration in all axes. Once the self-heating effect has ended and the algorithm has stored the information of this first segment, the heat source is started, leading to the second segment. The third segment is achieved after performing the rotation while keeping the heat source on. Finally, the heat source is turned off to reach the fourth segment. These segments and the information obtained from each of them is shown in Figure 7.

All data generated by the accelerometer during its calibration tests are logged in a file. This allows each test to be analyzed with external software and comparison of the results obtained using different techniques.

The duration of the calibration test depends on multiple factors, such as the heating power, the thermal inertia, and the self-heating delay. In practice, these tests take between 60 and 90 min.

### 4.4. Reference Tests

After the calibration tests, a set of six tests is performed for each DUT in a thermal chamber with larger thermal variations, −10 to 60 °C, and slower temperature steps: three hours each step and one hour each transition. Each of these six tests takes 24 h; therefore, each unit is tested for a total of 144 h in order to obtain its thermal calibration parameters. These tests are used as a reference to analyze the performance of the proposed fast technique.

### 4.5. Error Analysis

The performance of the techniques is analyzed by comparing them and the original data. This is achieved by studying the variability of the acceleration against the changes in temperature before and after thermal compensation. As a measure of performance, the typical acceleration variation in the temperature range studied (−10 to 60 °C) will be used.

The error is calculated as the average acceleration variation during temperature changes, that is, the difference between the average acceleration of one section and the next. This results in the average variation between the steps used; each step is interpolated to the full studied range to average them. Therefore, Equation (10) is used to calculate the error of each test, where *i* is each thermal section and *n* is the number of sections.

(10) $$E=\frac{\sum_{i=1}^{n-1}\left(\right|{\bar{Acc}}_{i}-{\bar{Acc}}_{i+1}\left|\cdot \frac{Full\;Range}{\Delta T_{i}}\right)}{n-1}$$

Note that if the average acceleration of the first and last sections are the same, Equation (10) is equivalent to calculating the difference directly between the highest and lowest average acceleration, as shown in Equation (11). Those accelerations should coincide with the steps with the highest and lowest temperatures, although the order may change.

(11) $$E=|{\bar{Acc}}_{Hot}-{\bar{Acc}}_{Cold}|$$

The full-scale error is calculated as the percentage that this error represents on a working scale of ±1 *g*, as shown in Equation (12).

(12) $$E_{FS}=\frac{E}{Full\;Scale}\cdot 100\%$$

## 5. Results

To ensure the functionality of this technique, two main aspects have to be analyzed. First, a comparison between the microcontroller algorithm and more powerful analysis is made. This aims to ensure that the self-calibration algorithm, able to run in MCUs with low resources, ends with the same results as more traditional analysis. Secondly, this fast technique is compared with the traditional compensation technique using six tests, longer temperature steps, and lower gradients.

**Figure 8:**
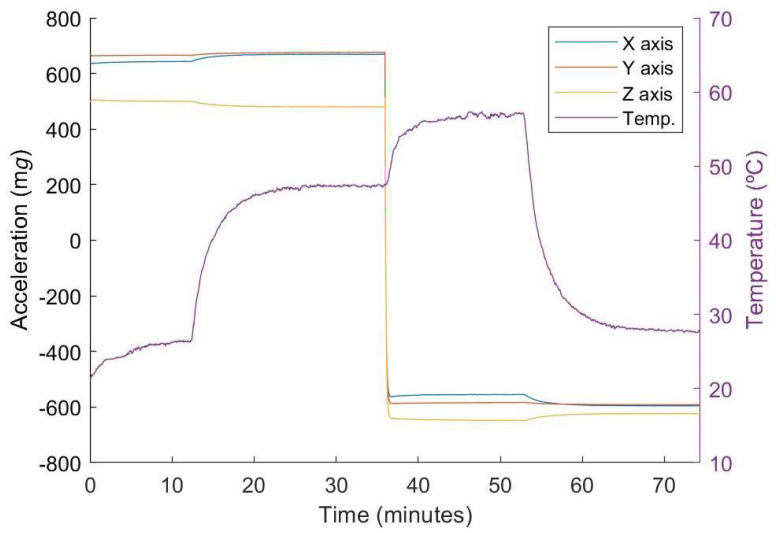
Acceleration and temperature values during the proposed calibration test.

First of all, the data logged during the fast calibration tests are analyzed. Data corresponding to one of these tests are represented in Figure 8. There are multiple characteristics that stand out in the figure. The four segments of the fast calibration tests required for the algorithm to work can be clearly distinguished in both the acceleration and the temperature data. The temperature also shows a thermal increase when the DUT changes orientation. This is related to the different position of the accelerometer, which is then above the heat source and, therefore, concentrating a greater quantity of heat, since the heating power remained constant.

### 5.1. Algorithm Analysis

**Figure 9:**
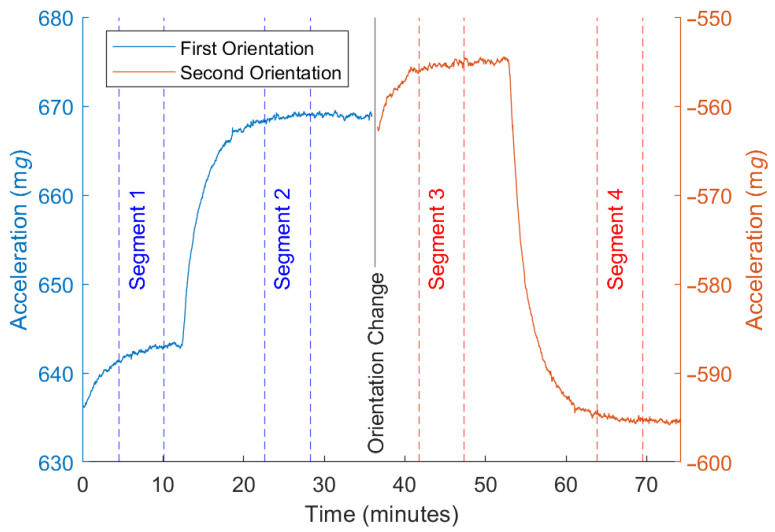
Segments analyzed by the algorithm during the calibration test.

Figure 9 shows in detail the X axis during both orientations of the calibration test. The segments logged by the algorithm in order to compute the thermal drift parameters have been highlighted. The drift caused by the self-heating effect is also visible at the beginning of this series.

As previously stated, this technique relies on the fact that the thermal drift for a specific orientation is linear. If the relation between both variables was not linear, two points would not contain enough information to characterize the thermal drift.

**Figure 10:**
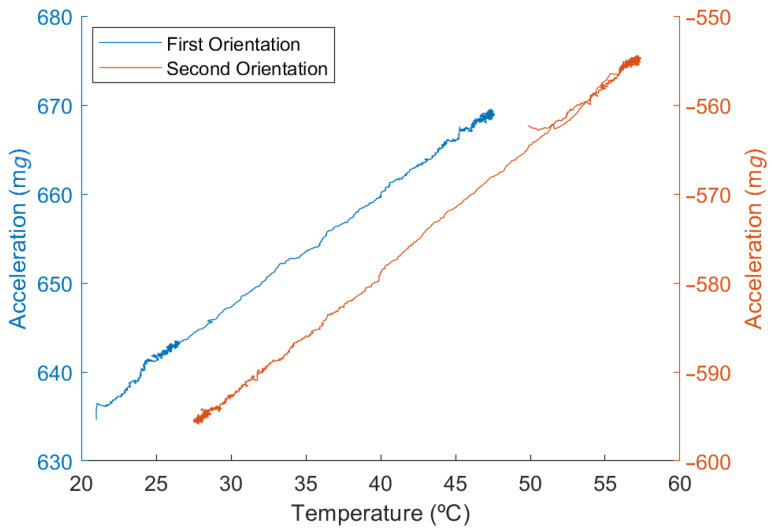
Acceleration vs. temperature in both test orientations.

This can be checked using a regression for each orientation of the test, not for the test as a whole, since the $TD_{i}$ varies as the orientation changes. The relation between temperature and acceleration for both orientations is shown in Figure 10. Two vertical axes with different values but the same scale are used to show both test sections simultaneously and how their slope is similar but not identical. It can also be seen that the temperature range is different in both sections of the test, as previously mentioned.

The slope of each series is the $TD_{i}$ for that orientation; the difference between the slopes in both orientations is caused by the TDSF. Having a small difference means that the TDB, which is related to the slope at 0 mg, affects the system in a more significant way than the TDSF, which is related to the change in slope according to the acceleration.

This linearity can be analyzed as the goodness of fit of a linear regression to the data. In both cases, the $R^{2}$ exceeds 0.999, meaning that the relation between both variables is linear, and therefore, two points are enough to obtain the relevant information. Due to having access to all the test data, it is also possible to compare the results provided by the MCU to those obtained with Matlab^®^ R2021a (MathWorks, Natick, MA, USA) using the same algorithm or using the regression with all the data. Table 5 shows the computed values for the $TD_{i}$ and the $Acc_{0}$, since the TDB and TDSF are computed in the same way with these data.

The results obtained by the MCU in real time using the proposed algorithm are similar to those obtained using the same data but more complex techniques. The differences are below 1%, while the required time per cycle for the algorithm to work averages only 267 μs (using the SAMD21G18A). Since it has an output data rate of 3.125 Hz, the algorithm requires approximately 800 μs every second, which is less than 0.1% of the time.

### 5.2. Technique Analysis

**Figure 11:**
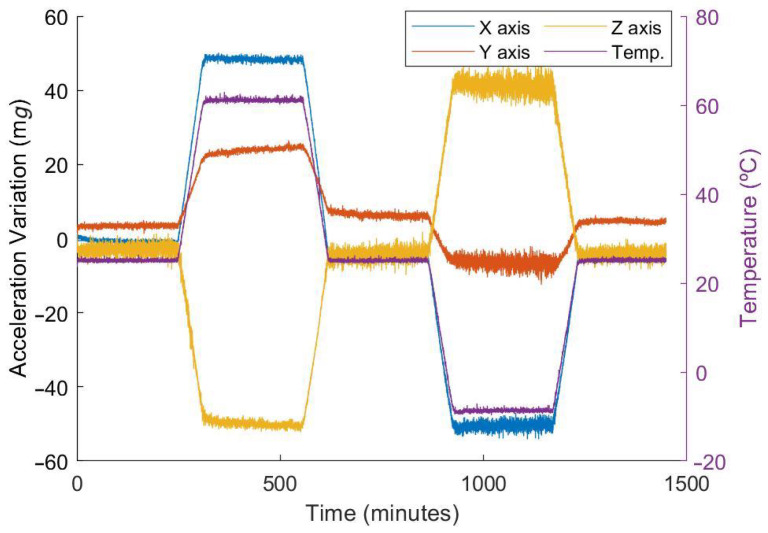
Acceleration drifts and temperature values during the calibration test.

The proposed fast technique can also be compared with traditional, slower, techniques. As previously stated, the slow calibration technique, used as a reference, consists of six independent tests. Each of them is performed by aligning one axis with the gravity vector, and it includes five thermal segments, each of them lasting three hours. The acceleration variations and the temperature profile logged during one of these tests is shown in Figure 11.

**Figure 12:**
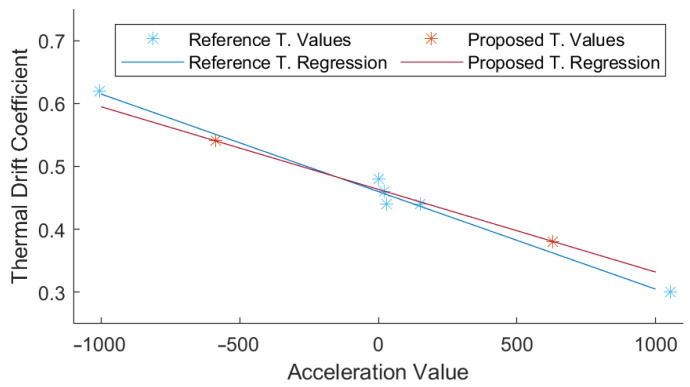
Data points and thermal drift coefficients regressions for the proposed and reference techniques.

Each test is analyzed with a regression and to obtain a $TD_{i}$ and a $Acc_{0}$, similarly to the fast technique. In this case, six different points are obtained for each DUT. A second regression is performed to obtain the TDB and the TDSF. The points obtained with both techniques, and the corresponding linear regression, can be represented simultaneously to show the differences between them, as shown in Figure 12.

**Figure 13:**
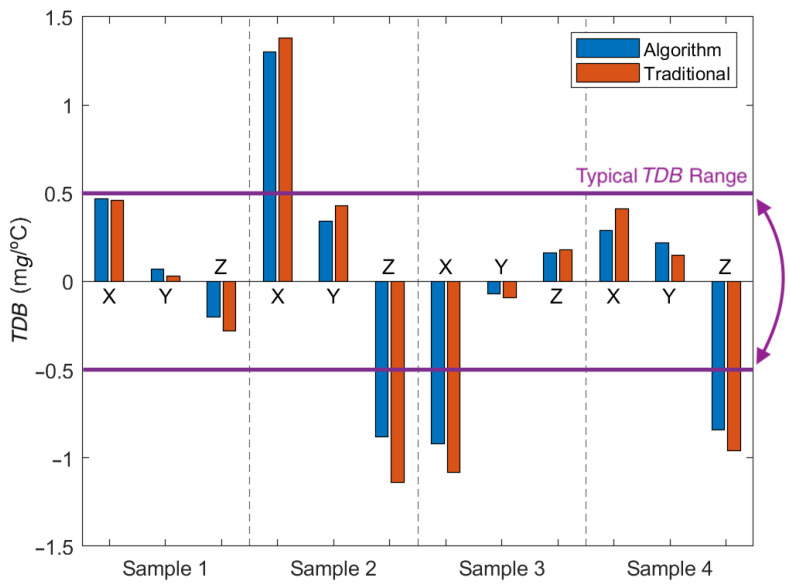
Comparison between the TDB values obtained with both techniques.

The TDB is the value that affects the thermal drift the most, with the TDSF generating small adjustments. A comparison between all TDB values obtained with the fast technique and the reference technique is shown in Figure 13. The average difference between both techniques is 0.09 mg/°C. The TDB values obtained fit previous studies, ranging between 1.4 and −1.2 mg/°C. The average magnitude is 0.55 mg/°C, which is very similar to the typical value provided by the manufacturer, 0.5 mg/°C. In fact, two-thirds of the TDB values obtained are lower than the manufacturers’ typical value, with two X axes and two Z axes exceeding the typical value of ±0.5 mg/°C. All the obtained TDB and TDSF values obtained are presented in Table 6.

### 5.3. Compensation

To check the actual performance this calibration technique offers in application, the slow tests are thermally compensated. The thermal chamber data are used as the reference for compensation. Equation (1) can be rearranged into Equation (13) in order to compensate for the thermal drift.

(13) $$Acc_{0}=\frac{Acc-TDB\cdot \Delta T}{1+TDSF\cdot \Delta T}$$

**Figure 14:**
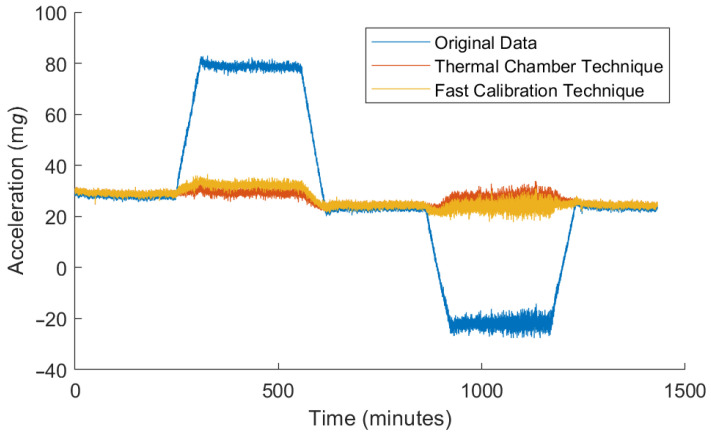
Thermal drift compensation with the parameters obtained in both cases.

Figure 14 shows one of the tests uncompensated and its comparison with the compensated data using both the proposed technique and the reference technique.

**Figure 15:**
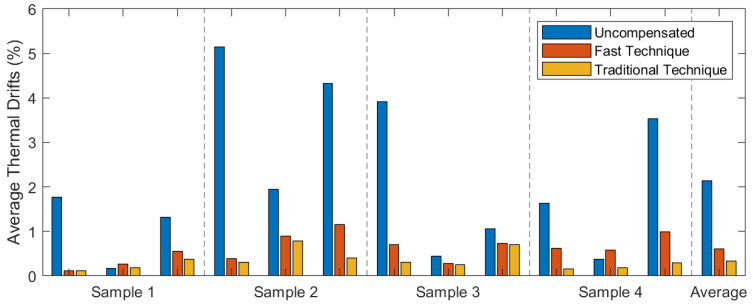
Average relative errors before and after compensation with both techniques.

Both techniques effectively reduce the thermal drift of the accelerometer. To quantify the improvement, the average drift between the different thermal steps is measured for all the slow tests, using Equations (10) and (12). The errors for each unit, relative to the full scale (±1 *g*), are shown in Figure 15 for all axes and samples. The maximum and average drifts in each case are shown in Table 7.

Before the compensation, the average drift during the tests is 42.7 mg with a maximum drift of 103 mg. This is mostly caused by the thermal drift of bias, which averages 0.61 mg/°C and reaches up to 1.4 mg/°C. The proposed calibration technique is able to reduce this drift to an average of 12 mg, the maximum being 23 mg. The average relative error is reduced from 2.14% to 0.61%, and all the studied axes, except for one, show a relative error lower than 1% after compensation.

## 6. Conclusions

Using the same data, the proposed algorithm obtains the same results as more complex computations. This allows for the calibration to be performed without complex equipment, which can lead to self-calibration and in-field calibration features for MEMS capacitive accelerometers.

Due to the low memory and computing requirements of the algorithm, it could be modified to allow slower in-field calibrations in parallel with other applications. This would allow each system to use real application data to adjust its calibration, which would result in a more consistent compensation of the thermal drift.

The proposed technique is able to effectively characterize the thermal behavior of MEMS capacitive accelerometers and compensate for its drifts. The average error during the tests is reduced to 28% of the original value. During the tests with thermal variations up to 70 °C, the average errors are reduced from 42 to 12 mg.

Due to the reduction of orientations and temperatures, the increase of the thermal gradient, and to carry out the computation in the microcontroller itself, the calibration can be performed in less than two hours, and since each unit computes its own calibration, multiple units can be calibrated at the same time using the same structure and thermal steps. This further reduces the time required for each calibration when multiple units are involved.

In comparison with other techniques, the one proposed in this work allows for a faster and easier calibration, since traditional methods require laboratory hardware or software, and their tests usually take longer than 20 h. In contrast, the main disadvantage of this technique may be its reduced study range, both in temperature and acceleration. This could lead to worse precision in the obtained parameters and, therefore, a reduced performance of the compensation.

It is difficult for any compensation technique to achieve a perfect thermal compensation. Even the reference technique used in this work, requiring six orientations and 24 h per test, has some residuals after the compensation. The relative error of the proposed algorithm only deviates from the reference a 0.3% with 70 °C thermal steps, while requiring less than 1.4% of the calibration time.

## Figures and Tables

**Table 1:** Matrix that contains the tests average accelerations.

Orientation	Segment 1	Segment 2	Segment 3	…	Segment N
1	$Acc_{11}$	$Acc_{12}$	$Acc_{13}$	…	$Acc_{1N}$
2	$Acc_{21}$	$Acc_{22}$	$Acc_{23}$	…	$Acc_{2N}$

**Table 2:** Final data obtained for each axis.

Orientation	$TD_{i}$	$Acc_{0}$
1	$TD_{1}$	$Acc_{01}$
2	$TD_{2}$	$Acc_{02}$

**Table 3:** Typical characteristics of the LIS3DSHTR MEMS accelerometer in the ±2 *g* range configuration.

Parameter	Typical Value
Resolution	16 bits
Sensitivity	0.06 mg
Output data rate	3.125 Hz to 1.6 kHz
Sensitivity change vs. temperature (TDSF)	0.01%/°C
Typical zero-*g* level offset accuracy	±40 mg
Zero-*g* level change vs. temperature (TDB)	±0.5 mg/°C

**Table 4:** Configuration of the studied units.

Register	Value (HEX)	Details
CTRL_REG4 (20 h)	17	ODR: 3.125 Hz. All axes active
CTRL_REG5 (24 h)	C0	Antialiasing: 50 Hz. FS: ±2 *g*.

**Table 5:** Data obtained with the three methods: Microcontroller Algorithm (MCU), Matlab^®^ Algorithm (Matlab), and MatLab^®^ Linear Regression (Regr.).

	Thermal Drift			Theoretical Acceleration		
Orientation	MCU	Matlab	Regr.	MCU	Matlab	Regr.
1	1.233	1.232	1.234	641.5 mg	641.5 mg	641.5 mg
2	1.388	1.378	1.394	−599.3 mg	−599.2 mg	−599.5 mg

**Table 6:** TDB and TDSF value obtained for all the samples and axes with both techniques.

	Proposed Technique						Reference Technique					
	TDB			TDSF			TDB			TDSF		
**Sample**	**X**	**Y**	**Z**	**X**	**Y**	Z	**X**	**Y**	**Z**	**X**	**Y**	**Z**
1	0.47	0.07	−0.20	−125	−38	0	0.46	0.03	−0.28	−30	−38	−58
2	1.30	0.34	−0.88	−113	0	−96	1.38	0.43	−1.14	−61	−30	−57
3	−0.92	−0.07	0.16	−167	−35	0	−1.08	−0.09	0.18	−4	−25	−56
4	0.29	0.22	−0.84	0	−200	−496	0.41	0.15	−0.96	−177	−169	−401

**Table 7:** Average and maximum deviations before and after compensations.

	Maximum Drift			Average Drift		
	Uncomp	Fast	Trad	Uncomp	Fast	Trad
*E* [mg]	103.10	23.11	15.72	42.77	12.18	6.85
$E_{FS}$ [%]	5.15	1.16	0.79	2.14	0.61	0.34

## Data Availability

Not applicable.

## Abbreviations

The following abbreviations are used in this manuscript:

MEMS	Micro-Electro-Mechanical System
TDB	Temperature Drift of Bias
TDSF	Temperature Drift of Scale Factor
MCU	Microcontroller Unit
DUT	Device Under Test
